# Editorial

**Published:** 1876-05

**Authors:** 


### Editorial.

### THE WORK OF STATE SOCIETIES.

The dental profession in every state should have a legal recognition and status, this would give strength of character and add prestige. Such legal enactments should be obtained, as would restrain those who ought to be restrained, and encourage and stimulate all honest and competent members of the profession. As yet but few states, five or six perhaps, have such laws. And these have in almost every instance been secured by the efforts of the state societies.

State societies can do very much in the way of modifying and regulating the ethics of the profession. Something has already been done in this particular, but considered as a whole only a beginning has been made.

But the question may occur, what can a state society do in this respect? It is within the province of, and entirely feasible for any state dental society to gain such strength, and establish such a character as to exert and command the regard and respect of all, yes to the extent that no competent member of the profession can afford to remain separate from it. Association in co-operative effort for mutual improvement and effecting the greatest good, always receives approval and respect.

The dentists in every state in the union ought to be organized and stimulated up to the point of taking up and carrying on important enterprises looking to development and progress. There should be in every state a thoroughly organized society, that should embrace the whole of the profession in the state, at least all that is worth any thing, and every man should be made available and efficient for public good in some particular. Then the state society might have the supervision and fostering care of local societies in its territory, and organize them in all localities where there is a sufficient number to warrant it; let competent men be assigned to this work in the various districts, let reports be made by all local societies to the state societies at their annual meetings. Let each state society do whatever it can, for the encouragement of local societies. The benefits derivable from local association are many, and can hardly be estimated; much can be done by them that is wholly impracticable in larger bodies, only by such effort can a thorough acquaintance with each other be effected, and a proper harmony and co-operation be secured; and in addition to this regular and systematic study of special branches can be taken up and pursued with great profit. Clinic work can be far better pursued in such meetings than when larger numbers are present.

Our State Societies may influence and give direction, to a great extent, to the educational interests of those who are to be members of the profession in the future.

Every State Society should send delegates to other state societies, and in that way, whatever of development was made in any one, would thus have a far wider influence; and greater uniformity in modes of action and procedure would be secured.

In states where there are no colleges, it would be well for societies to establish collections of interesting subjects and materials, this would be especially feasible, where the society is always held at the same place.

We have here only indicated a part of the subjects that should receive the attention of our state societies.

We regard these organizations, as one of the grand instrumentalities, for the elevation of the profession; and we trust that the time is near at hand when they will be made far more effective than they have hitherto been,

### MICRO-PHOTOGRAPHS.

We have just received a package containing about twenty micro-photographs, from Dr. Geo. B. Harriman, of Boston. They are photographs of blood, showing the disks and corpuscles beautifully. Blood of man and various animals, viz: the ox, the horse, the pig, the sheep, the rabbit, the rat, the trout, the squirrel, the mouse and the dog is represented. The difference and peculiarities of each being clearly distinguishable. Cataract, and other affections of the eye are also represented.

One of the pictures is of the blood from a consumptive patient; another from one having syphilis; these show marked difference not only from each other but from healthy blood; whether these peculiarities, are truly characteristic, and would be uniformily found; or whether they are only accidental and not clearly indicative of the conditions named, is questionable, and will require a far greater range of experiments to determine. An interesting fact in connection with these photographs is that they were all taken with a $\frac{1}{50}$th objective, except one taken with a $\frac{1}{75}$th. They are remarkably well defined and brought out in every respect considering the high power of the glasses used.

Dr. H. is an indefatigable worker with the microscope, and seems determined to make it something more than a play thing, or mere matter of amusement.

### COMMENCEMENT.

The first annual commencement of the Dental College of Michigan University was held March 29th, at 10 o'clock a. m., in connection with the commencement of the Medical College.

The degree of D. D. S. was conferred on D. C. Hauxhurst, W. H. Jackson, G. E. Post, L. L. Davis, of Michigan, and R. H, Tremper of Ohio

To these gentlemen as well as others this was an interesting occasion, they being the first graduates from this recently organized Institution.

This College promises well for the future; in certain directions it promises equal, if not superior facilities, to any similar institution, and this is especially true of the field for clinical teaching, no better could be asked; and as for the didactic teaching, in the various foundation branches, the reputation and ability of Drs. Ford, Palmer, Douglas, Dunster and Maclane is a sufficient guarantee.

We hope that ere long the course in this College will be nine months, instead of six as now.

The Dental Profession of Michigan may well congratulate themselves, on the work thus far accomplished in reference to special education.

---

### DIED

At Mansfield, Ohio, April 17th, 1875, Dr. M. DeCamp, of consumption, from which disease he had been a sufferer for more than a year before his death. Dr. DeCamp as a dentist, standing high in the profession; as a man of sterling purpose and strict integrity and as a Christian gentleman, seems to deserve more than a passing notice. The writer of this associated with him for nearly twenty years in the practice of the profession, claims some right to speak of the virtues of our Brother who has passed away. Dr. DeCamp was born in Washington County, Pennsylvania. October 10th, 1816. In the fall of 1827 his father, with his family, moved to Morrow County, Ohio. As a lad, Dr. DeCcamp had his part to perform incident to the establishing of a home in a new country. But in the mean time, by the light of the fire in the old fireplace, he acquired sufficient book knowledge to enable him to teach. In this he was successful and the Fall of 1842 found him in charge of what was then known as the preparatory department of Delaware University, which embraced almost every branch except the dead languages. His duties were arduous. The result was a partial loss of voice which compelled him to abandon his chosen profession. He had by the faithful discharge of his duties, built up more than a local reputation, as was shown by a proposition tendered to him from the trustees of a Tennessee college to act as its president, with a salary of $1,200 the first year, and an annual addition of $200 until it reached $2,000 a year. This was a large salary, when we consider that Bishop Thompson who was then president at Delaware, received but $600.

He refused this offer partly on account of his want of health, and partly on account of Tennessee being a slave state. Soon after this he removed to Mt. Vernon, Ohio, where he married Almena H. Winters, in the fall of 1844. After a thorough course of preparation in the office of Dr. Reeve, then one of the best dentists in the State, he removed to and commenced the practice of dentistry in Mansfield, Ohio. He was eminently successful as a practitioner. His business constantly increased, until the year 1854, when he found it necessary to secure help to aid him in his business, and the writer became associated with him. Dr. DeCamp was possessed of many excellent qualities of head and heart. Genial in disposition, affable in manners, strict but generous in all his dealings with his fellow men. Kind of heart; the deserving poor seldom appealed to him in vain. Himself, in his early days, an educator, he took a lively interest in all things tending to promote the elevation of his chosen profession. He gave liberally of his means for educational purposes, and was one of the early patrons of the Ohio Dental

College, assisting in planting a firm foundation and for a number of years a member of its Board of Trustees. He held the degree of D. D. S., from this institution. He was always careful in the selection of young men who were to become his pupils, and of those who left his office to engage in the practice, may be found to-day not a few who in intelligence and as first class operators are the equal of any young practitioners in the country. On the passage of the law regulating the practice of dentistry in the State of Ohio, he was chosen a member of the Board of Dental Examiners and occupied that position for several years. He was a member of the American Dental Association as well as the Ohio Dental Society, and until prevented by his last illness, was a regular attendant on the sessions of these bodies.

He had so lived that when the grim messenger of Death appeared, he had no terrors for him. Patient and cheerful to a marked degree, he had little to do save to wait for the end to come. Happy may we all feel if, like him, we can use in sincerity this, almost his last earthly saying: "My life is not a failure, no, it is a glorious victory. I have gained eternal life." C. R. T.

CINCINNATI, O., April 28, 1876.

### IN MEMORIAM.

At a special meeting of the Central Ohio Dental Society, a committee was appointed to draft resolutions of respect, and presented the following:

WHEREAS, Dr. M. DeCamp, our beloved President, has been removed from our midst by death. Therefore

*Resolved*, That in his death our Society has lost a most active and useful member, and the profession one of its most earnest and influential supporters, and society one who was always ready with words of cheer and comfort and with money to assist the needy. One whose every action endeared him to all with whom he became associated.

*Resolved*, That the members of this Society tender their sympathy to the relatives and friends of our departed brother.

*Resolved*, That a copy of these resolutions be presented to the family of the deceased, and that they be published in the Mansfield papers and the DENTAL REGISTER.

L. W. NEVIUS,<br>
T. S. SEELY,<br>
H. J. CRESSINGER, } Committee.

### AMERICAN DENTAL ASSOCIATION.

The American Dental Association holds its annual meeting in Philadelphia, beginning on the 1st Tuesday of August next.

The indications are that there will be a very large and interesting meeting. Exercises and reports befitting the occasion will doubtless be presented.

There will be present members of the profession from other countries. And if there should be any considerable number of these an international Association or Congress, might with propriety and profit be formed.

We would suggest that the Dentists of this country and all others too, make their visits, so far as possible, at the time of this meeting.

